# Severe Scurvy After Gastric Bypass Surgery and a Poor Postoperative Diet

**DOI:** 10.4021/jocmr726w

**Published:** 2012-03-23

**Authors:** Esben P.K. Hansen, Carsten Metzsche, Emil Henningsen, Palle Toft

**Affiliations:** a Department Anaesthesiology and Intensive Care, Odense University Hospital, 5000 Odense C, Denmark; b Department Dermatology, Odense University Hospital, 5000 Odense C, Denmark

## Abstract

After bariatric gastric bypass surgery patients are at risk of developing micronutrient deficiencies. If gastric bypass surgery is followed by a vitamin deficient diet the patients have a risk of developing vitamin-C deficiency. When spontaneous ecchymosis is observed in the skin, in at-risk patients, scurvy must be considered. When treated with large doses of vitamin-C the symptoms of scurvy rapidly improve even if the patient has developed multiple organ dysfunction syndrome.

**Keywords:**

Scurvy; Gastric bypass surgery; Multiorgan dysfunction

## Introduction

When humans are deprived of vitamin C the first symptoms of scurvy appear 29 - 90 days later as demonstrated by Hodges et al. [1]. Scurvy has historically been described among sailors deprived of vitamin-C on long journeys. In an elegant study, ship surgeon J. Linn demonstrated that citrus fruit was effective in preventing scurvy. While scurvy still occurs in the third world it is extremely rare in developed countries. When scurvy is observed in developed countries it is often limited to certain at risk populations. One such at risk population comprises patients exposed to gastric bypass surgery. Following bariatric gastric bypass surgery the patients are at risk of developing a deficiency of especially fat-soluble vitamins [2, 3]. If bariatric patients after gastric bypass surgery receive an insufficient diet the risk of vitamin-C deficiency will also exist. We describe a case of severe scurvy after bariatric bypass surgery combined with an insufficient postoperative diet. Our case shows that even in cases of severe scurvy mimicking multiple organ dysfunction syndrome the condition is reversible and easy to treat with vitamin-C.

## Case Report

A 45 year old woman had a bariatric gastric bypass surgical operation. A few years before the operation she had a weight of 192 kg (BMI 65). Over a two years period she was able to reduce her weight to 149 kg. This preoperative weight of 149 kg was stable over the last four months before the gastric bypass operation. She had a medical history of insulin-dependent diabetes mellitus and severe psoriasis. She had also been hospitalised with erysipelas and ulcers on the lower extremities a few years prior to having gastric bypass surgery.

Though she received postoperative advice from a dietician about the importance of eating a vitamin rich diet, she continued with an insufficient diet in order to obtain further weight loss. Three months postoperatively she had lost further 20 kg in weight. At the same time she began to develop painful swelling, bruising and small ulcers on the lower extremities. She was hospitalised at the local hospital with fever one week after these symptoms appeared. Within the next 24 hours she developed purpura on the lower extremities and a sepsis like condition. Due to this she was transferred to Intensive care unit (ICU) at Odense University hospital. Within days she developed multiple organ dysfunction syndrome with cerebral, respiratory, circulatory and renal failure. It was necessary to intubate and ventilate the patient and renal failure was treated with continuous renal replacement therapy (CRRT). To restore the circulation it was necessary to treat the patient with a large amount of intravenous fluids and vasopressor agents. Infected leg ulcers were suspected to be the focus for sepsis. After appropriate samples of blood and secretion were taken for cultures, broad-spectrum antibiotic was initiated. No bacteria occurred in the cultures and the patient showed no signs of improvement on antibiotic therapy. In contrast the changes in the skin progressed with increasing erythema, and ecchymosis forming a painful, confluent purpuric plaques and bullae. Minimal trauma to the skin resulted in petechiae and confluent purpuric plaques. It was estimated that 30% of the skin was affected by confluent purpuric plaques. As a result of these haemorrhagic bullae the patient was continuously bleeding from the skin. On the worst days the blood loss from the skin was estimated to be 61 per day. In addition to blood loss the patient lost a large amount of fluid from the skin-estimated on the worst days to be 201 per day. The patient’s lips were covered with haemorrhagic crust but no major gingival bleeding was observed. Skin biopsy was performed. This biopsy showed diffuse extravasation of erythrocytes but no hyperkeratosis or “corkscrew hair”. The subdermal tissue was vital. A biopsy from the bone marrow was without any sign of malignancy.

**Figure 1:**
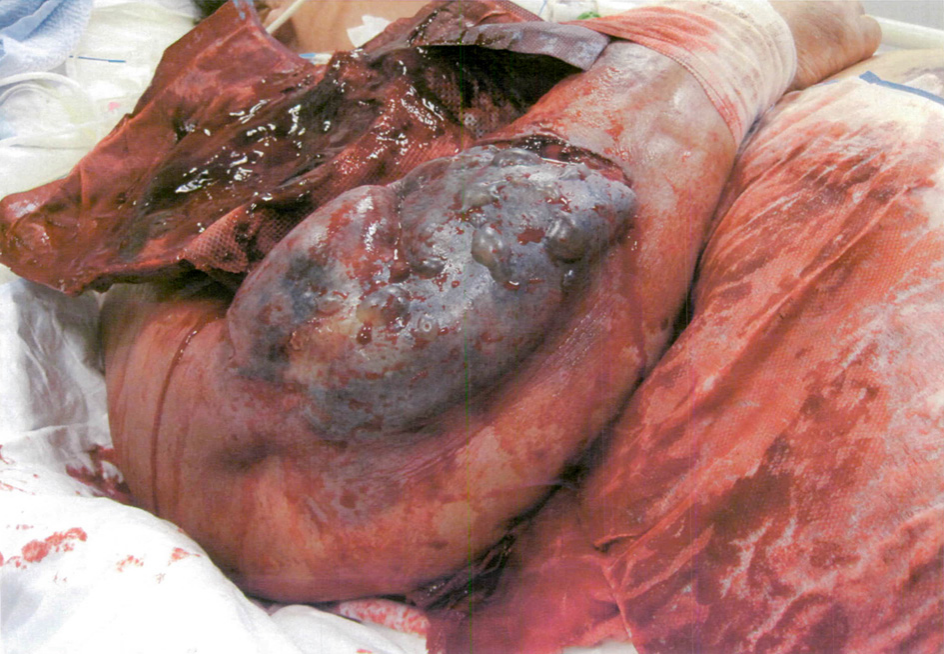
Illustrates the confluent purpuric plaques and bullae.

Over the three weeks in the ICU it became obvious that the skin changes and the massive loss of blood and fluid from the skin were not in accordance with a diagnosis of sepsis. It was proposed that the massive bleeding from the skin might be due to scurvy. A blood sample for the analysis of serum-ascorbic acid levels was taken and the patient was treated with enteral ascorbic acid at a dose of 2 g per day. The blood sample showed a serum-ascorbic acid concentration of 6.6 (normal range 26 - 85 μmol per l). After two days of vitamin-C treatment the patient’s condition began gradually to improve. The skin changes gradually disappeared, haemodynamics improved, the patient regained her renal function and could be weaned from the ventilator. A few weeks after the start of ascorbic acid treatment the patient could be transferred to the medical ward.

## Discussion

Following bariatric gastric bypass surgery the patients are at risk of developing micronutrient deficiency. Patients are especially at risk of developing deficiency of fat-soluble vitamins - in particular vitamins A and D but also E and K [2-4]. These patients are also at risk of developing deficiency of vitamin B12, iron and zinc. Finally, as illustrated by our case, gastric bypass patients are also at risk of developing vitamin C deficiency if the operation is combined with a poor diet.

Scurvy is a rare disease and is confined to certain at-risk groups. In the developed countries these at-risk groups consist of patients with poor nutritional health such as mentally retarded persons, alcoholics with poor nutrition, elderly persons living alone and patients who have undergone gastric bypass surgery.

Hodges et al. showed that non specific symptoms of vitamin C deficiency develop 4 - 6 weeks after the start of vitamin C depleted nutrition. More severe symptoms of scurvy develop after 1 - 3 months. The study by Hodges et al. [1] showed that haemorrhage was the first symptom to appear; ecchymosis was observed in more than 80% of the patients. This is in accordance with the present study as haemorrhage and ecchymosis complicated by loss of fluid were the most prominent symptoms. Our patients developed severe scurvy three months after bariatric gastric bypass surgery combined with vitamin-C poor diet. This timeframe is also in accordance with the observation made by Hodges et al. [1]. While others have described severe periodontal disease as a prominent marker of scurvy our patient had no severe bleeding from the mucosa in the oral cavity [5].

The recommended daily dose of vitamin-C for adults is 45 - 99 mg. From the day of admission on the ICU our patient received enteral nutrition by a nasogastric tube. The daily amount of vitamin-C provided by this enteral feed was 133.4 mg. Though the patient’s daily requirement of vitamin-C was being met this was not enough to treat a severe case of scurvy. This was also attributed to the simultaneous use of CRRT. It is well known that water-soluble vitamins including vitamin-C are lost during CRRT treatment. The substantial transcutaneous fluid loss, bleeding and to a lesser extent diabetes, might also have influenced the low concentration of vitamin-C in the blood.

Our case shows that vitamin-C deficiency should be considered in at-risk patient groups including patients post bariatric gastric bypass surgery who present with petechiae or spontaneous ecchymosis of the skin. Scurvy should also be considered in patients with spontaneous ecchymosis and multiple organ dysfunction syndrome without an obvious underlying disease. Untreated scurvy is fatal. Scurvy is however an easy condition to treat with the rapid resolution of symptoms after treatment with vitamin-C has been initiated. This case demonstrates that even when symptoms of scurvy have progressed into multiple organ dysfunction syndrome the disease will resolve dramatically and quickly when treated with vitamin-C.

In conclusion, bariatric gastric bypass patients are at risk of vitamin C deficiency if not supplemented with vitamin C. In patients with spontaneous ecchymosis in the skin and nutritional problems scurvy ought to be considered. Scurvy, even with progression to multiple organ dysfunction syndrome improves rapidly when treated with large doses of vitamin C.

## References

[1] Halligan TJ, Russell NG, Dunn WJ, Caldroney SJ, Skelton TB (2005). Identification and treatment of scurvy: a case report. Oral Surg Oral Med Oral Pathol Oral Radiol Endod.

[2] Hodges RE, Hood J, Canham JE, Sauberlich HE, Baker EM (1971). Clinical manifestations of ascorbic acid deficiency in man. Am J Clin Nutr.

[3] Schweitzer DH, Posthuma EF (2008). Prevention of vitamin and mineral deficiencies after bariatric surgery: evidence and algorithms. Obes Surg.

[4] Aasheim ET, Bjorkman S, Sovik TT, Engstrom M, Hanvold SE, Mala T, Olbers T (2009). Vitamin status after bariatric surgery: a randomized study of gastric bypass and duodenal switch. Am J Clin Nutr.

[5] Minambres I, Chico A, Perez A (2011). Severe Hypocalcemia due to Vitamin D Deficiency after Extended Roux-en-Y Gastric Bypass. J Obes.

